# Quantum hyperparallel algorithm for matrix multiplication

**DOI:** 10.1038/srep24910

**Published:** 2016-04-29

**Authors:** Xin-Ding Zhang, Xiao-Ming Zhang, Zheng-Yuan Xue

**Affiliations:** 1 Guangdong Provincial Key Laboratory of Quantum Engineering and Quantum Materials, and School of Physics and Telecommunication Engineering, South China Normal University, Guangzhou 510006, China

## Abstract

Hyperentangled states, entangled states with more than one degree of freedom, are considered as promising resource in quantum computation. Here we present a hyperparallel quantum algorithm for matrix multiplication with time complexity *O*(*N*^2^), which is better than the best known classical algorithm. In our scheme, an *N* dimensional vector is mapped to the state of a single source, which is separated to *N* paths. With the assistance of hyperentangled states, the inner product of two vectors can be calculated with a time complexity independent of dimension *N*. Our algorithm shows that hyperparallel quantum computation may provide a useful tool in quantum machine learning and “big data” analysis.

Quantum algorithms^1^ are believed to be able to speedup dramatically for some problems over the classical ones. Ground breaking work include large integer factoring with Shor algorithm^2^, Gorver’s search algorithm^3^,^4^,^5^, and linear system algorithm^6^,^7^. Recently, quantum algorithms for matrix are attracting more and more attentions, for its promising ability in dealing with “big data”. For example, quantum speedup for linear system equation^6^,^7^, max-min matrix product^8^, Boolean matrix product^8^,^9^,^10^ have been studied.

Matrix multiplication is one of the most important operations for matrix as many other problems can be reduced to it, e.g., determinant and matrix inverse^11^. Up to now, only the algorithm for matrix product verification has been proposed^12^, i.e., to verify whether *AB* = *C* or not. The algorithm is based on quantum random walk and has a time complexity *O*(*N*^5/3^), which is better than the best known classical algorithm that runs in time *O*(*N*^2^). However, quantum algorithm for matrix multiplication without previous knowledge about the results has not yet been presented.

Swap test is a procedure that can determine the overlap of two quantum states, which is first introduced for quantum fingerprinting^13^,^14^. By entangling the tested system with an ancillary qubit, one can estimate the inner product of two different states by measuring the ancillary qubit repeatedly. Recently, in ref. 15, the authors proposed an algorithm for quantum machine learning based on swap test, which is exponentially faster than classical algorithm. The key step of the algorithm is mapping the *N*-dimensional vectors to a log_2_ *N* qubits state, then estimate the distance of two normalized vectors with swap test. An experiment in photonic system is also performed to prove its validity^16^. Those work show that swap test can play an important role for quantum algorithm.

However, for photonic systems, the preparing of many-photon entangled state becomes increasingly difficult for very large *N*, i.e., both generation rate and state fidelity will drop dramatically^17^,^18^,^19^,^20^. An alternative way is using hyperparallel quantum computation (HPQC)^21^,^22^,^23^. In HPQC, two or more qubits are encoded in different degree of freedom of a single source, which is called ‘hyperentanglement’. It requires much less resource and can avoid the infidelity problem during the multipartite entangled state generation process. Some important works in hyperentanglement have been reported. For example, the generation of hyper entangled states (HES) with very large dimension has been demonstrated experimentally^24^,^25^. The first complete hyperentangled Bell state analysis protocol was proposed in 2010^26^. Recently, the first experiment of quantum teleportation of multiple degrees of freedom of a single photon in polarization and orbital angularmomentum with linear optics was performed^27^. Based on the robust entanglement encoded in other degree of freedom such as spatial modes or time-bin, it is shown that it can be used to perform the deterministic entanglement distillation or purification^28^,^29^.

Here, we present a hyperparallel algorithm for matrix multiplication based on swap test. We show that besides reducing the required resource, HPQC also leads to significant speedup. In our algorithm, both the polarization degree of freedom and the spacial degree of freedom of the single photon are used. By introducing an extra degree of freedom, we do not need to prepare multipartite entanglement cluster states. Instead, an *N* dimensional vector is represented by only a single source, and the information of each element are mapped to the spacial degree of freedom. Therefore, for square matrix multiplication, our algorithm takes time *O*(*N*^2^*ε*^−2^ log_2_ *η*^−1^), where *m* and *ε* will be defined in the following . In comparison, the best known classical algorithm given by Williams takes time *N*^2.372 ^30, while the non-hyperparallel quantum algorithm takes time *O*(*N*^2^ log *N*). The speedup of HPQC comes from the state preparation procedure. For HES, preparing an *N* dimensional state takes time O(1), while conventional quantum algorithm takes time log(*N*). Our algorithm includes the output of all elements of the results. Since printing out *N*^2^ numbers takes time *O*(*N*^2^), our algorithm reaches the lower bound of time complexity for matrix multiplication^30^.

## Result

### The algorithm

Suppose we have two real number matrices


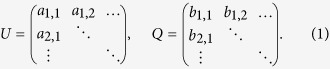


We define the vectors 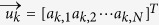, and 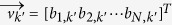. Matrix multiplication is to calculate *W* = *UQ*, where 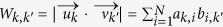.

Here, we represent the two level degree of freedom by alphabet *H* and *V*, and the paths degree of freedom by numbers 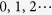. We begin with preparing an entangled state with respect to the two level degree of freedom


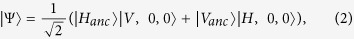


where |*H*(*V*), 0, 0〉 represents a tested source that is in path |0, 0〉. The first number denotes stem paths, while the second number denotes branch paths. The stem path of *k*(*k*′) > 0 represents the vector with label 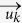 or 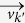 (*k* and *k*′ represent two different paths), and the branch path *i* represents different elements of the vector.

First, to calculate *W*_*k*,*k*′_ with particular accuracy, we apply a series of operations on the space expanded by paths and two level degrees of freedom (see the method section for details), which transform the state |Ψ〉 to


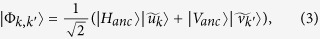


where


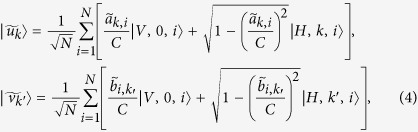


where 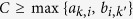 is a constant ensuring that the probability amplitudes of each paths will not exceed 1; 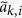 and 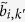 are the approximation of *a*_*k,i*_ and *b*_*i,k*_, i.e., 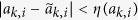, 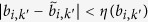. *η* (*a*_*k*,*i*_) and *η* (*b*_*i*_,_*k*′_) denote errors that are introduced by the oracles, which map the information of vectors to the states. The errors satisfy 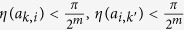, where *m* is the steps required for the oracle. Given the required accuracy, the steps of obtaining 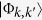 are independent to *N*.

The second terms of Eq. (4) are orthogonal to each other because they are in different stem paths *k* and *k*′. So the inner product of two states is simplified as


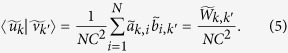


Here, 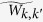 are the approximation of *W*_*k*,*k*′_, which satisfy


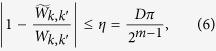


with 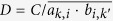 and D is the ratio of the mean of 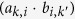 to *C*. To reach the accuracy *η*, the oracles require *O*(*m*) or *O*(log_2_ *η*^−1^) operations.

Next, to read out the inner product, we project the ancillary qubits to state 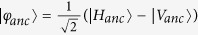. If the success probability of the projection is *p*_*k*,*k*′_, the element 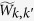 can be expressed as^15^


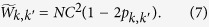


Experimentally, the probability *p*_*k*,*k*′_ can be obtained approximately with repeated measurements. For each element of *W*_*k*,*k*′_, we repeat the initialization, transform of tested qubit and measurement procedure for *T* times, among which there are 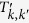 times of successfully projection. The probability can be approximated as 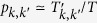. Therefore, we can define


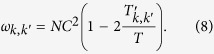


In this way, the error satisfies


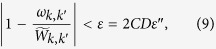


where 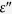 is the statistic error of *p*_*k*,*k*′_


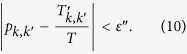


To reach the accuracy 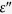 for *p*_*k*,*k*′_, or *ε* for 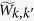, the repeating time is 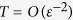. The total accuracy of *ω*_*k*,*k*′_ respected to *W*_*k*,*k*′_ can be given as


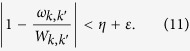


The time complexity of calculating *W*_*k*,*k*′_ is 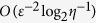. Since there are *N*^2^ elements to be calculated, the total time complexity of matrix multiplication is 
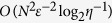
. We uses totally 2*N*^2^ oracles, while each oracle contains *O*(*m*) quantum gates. Therefore, the spacial complexity is *O*(*N*^2^*m*) or *O*(*N*^2^ log_2_*η*^−1^).

In summary, the procedure of calculating *W*_*k*,*k*′_ includes the following steps:

- Define the measuring time T, initially *T*′ = 0, *l* = 1;
- Initialize the state in |Ψ〉;
- Transform the state to |Φ_*k*,*k*′_〉;
- Perform the projection measurement at ancillary qubit, if success, *T*′ = *T*′ + 1;
- If *l* < *T*, *l* = *l* + 1,return to step 1; if *l* = *T*, output 1 − 2*T*′/*T*.

### Further speedup

At this stage, we present a further speedup of the algorithm, which allows one to calculate *N* numbers of *T*_*k*,*k*′_ synchronously. Previously, only two registers work at the same time, while other registers wait for the query. In fact, this waste can be avoided. We show that by sacrificing the spacial source, that is, increase the number of entanglement sources, quantum gates and the measurement devices, the algorithm can be speeded up dramatically by activating all registers at the same time.

We begin with preparing *N* pairs of entangled states 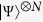, where 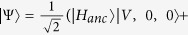

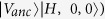
. As the procedure in the above section, we apply the separation operation to each qubit


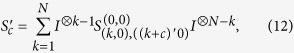


where integer 0 ≤ *c* < *N* is a constant for a particular loop of operation and (*k* + *c*)′ represents the register storing vectors 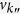 of matrix *V* with 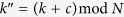. The state becomes


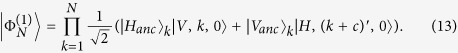


The *k*^*th*^ entanglement pair is now directed to the register *k* and (*k* + *c*)′ for calculating the element *W*_*k,k*+*c*_. Obviously, all 2*N* registers have been activated, and will process at the same time. We apply operation 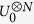 on the state and then direct the *i* branch path of the *k*^*th*^ qubit to oracle 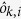 and 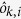. Finally, we obtain the state


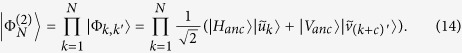


Next, we perform the projection measure of all *N* ancillary qubits onto the state 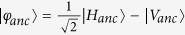. All values of 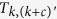 for a particular *c* can be obtained at the same time, and thus all values of 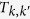 takes time *O*(*N*), instead of *O*(*N*^2^).

However, if we only speed up the calculation of *T*_*k*,(*k*+*c*)′_, the time complexity can not be reduced. The reason is that calculating *W*_*k*,*k*′_ from *T*_*k*,*k*′_ still needs *O*(*N*^2^) steps in classical computer, and the printing out of *N*^2^ still takes *O*(*N*^2^) steps. On the other hand, although the required sources increase, it is still much less than the required number of oracles *O*(*N*^2^). Therefore, the spacial complexity does not increase.

In conclusion, we have presented a hyperparallel algorithm for matrix multiplication with arbitrary high accuracy. Our work shows that besides reducing the spacial sources, HPQC can also speedup the algorithm. We show that the time complexity of swap test is independent of the vectors’ dimension. Therefore, related problems that can be solved by swap test, like quantum machine learning^15^,^16^, norm, etc., can also be speeded up with HPQC. In our algorithm, the time complexity respected to accuracy is restricted by the oracle. With a more optimized design of the oracle, it is possible to further speed up the algorithm. HPQC avoids the difficulties in preparing multipartite entanglement cluster states. So it may has superiority in both required sources and time complexity. Our work provides a new powerful tool for manipulating “big data” with quantum computer.

## Method

### The transform to |Φ_*k*,*k*′_〉

**Figure 1:**
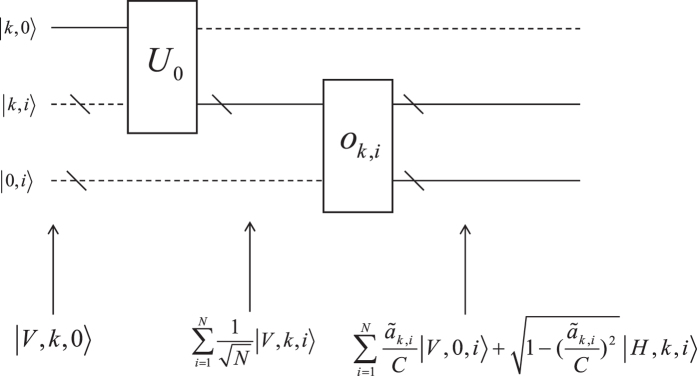
Quantum circuit of realizing the transformEq. (4). ‘/’ denotes a bundle of path. Dash line represents that the amplitude of such path is 0.

The transform Eq. (4) is the most critical part of our algorithm. As shown in Fig. 1, we now elaborate how to realize this procedure. To begin with, we define a separation operation


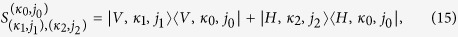


which separates states |*H*〉 and |*V*〉 of the tested source into different paths. For photonic quantum computation, such separation operation can be realized easily with PBS, which allows horizontally polarize light to pass through and reflect vertically polarized light.

The first step is implementing operation 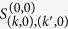 to the tested source. The state turns to


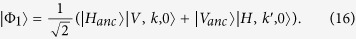


Then, we guide the tested sources to two different paths. Both paths connect to a register, which stores the information of vectors 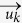 or 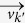. In registers we perform an operation *U*_0_ on the branch path degree of freedom


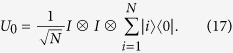


In this case the source is prepared as an equally distributed superposition state of branch paths


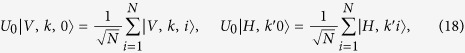


where |*k*, *i*〉 (|*k*′, *i*〉) corresponds to the elements *a*_*k*,*i*_ (*b*_*i*,*k*′_) of the vectors 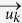 (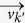). Next, paths |*k*, *i*〉 and |*k*′, *i*〉 are directed into oracles 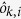 and 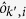, which control the rotation of the input state around axis-y in the space spanned by {|*H*〉, |*V*〉}. Generally, the oracles give transforms


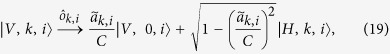


and


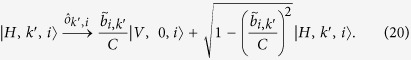


Finally, |*H*〉 and |*V*〉 are seperated to different paths by a separation operation, which lead the total output state to Eq. (4). For preparing state Eq. (13), we query all oracles synchronously. Therefore, the query complexity of the state preparation is independent of the dimension *N*.

### The oracle

**Figure 2:**
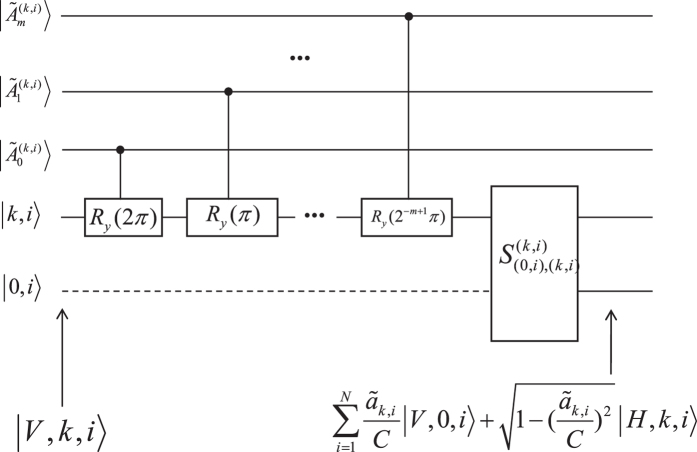
The realization of oracle 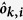. 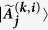 denotes work qubit. Dash line represents that the amplitude of such path is zero.

We now elaborate how to realize the oracles 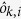 and 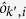 with arbitrary small error 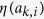 and 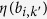. The quantum circuit of the oracle 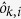 is given in Fig. 2. Each oracles 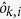 or 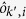 contains *m* + 1 control qubits 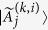 or 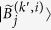 (0 ≤ *j* ≤ *m*). 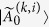 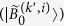 controls the sign of elements *a*_*k*,*i*_ (*b*_*i*,*k*′_), while other qubits controls the magnitude of *a*_*k*,*i*_ (*b*_*i*,*k*′_). The larger *m* is, the more accuracy the oracles are. Each work qubit can be on state |0〉 or |1〉.

First, we rewrite *a*_*k*,*i*_ and *b*_*i*,*k*′_ as


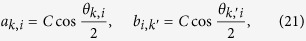


where *θ*_*k*,*i*_ and *θ*_*k*′,*i*_ can be expressed in terms of infinite series as


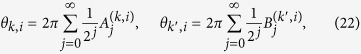


where 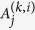 and 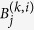 can be either 0 or 1. For 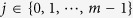, we prepare the state of 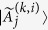 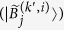 in a way that 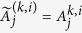 (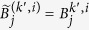). Then, we define angles 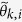 and 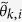, which are represented by control qubits:


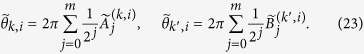


As can be seen, they are the approximation of *θ*_*k*,*i*_ and *θ*_*k*′,*i*_. The error between them satisfies


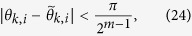


which can be infinitely small for infinitely large *m*, 
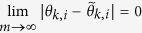
. The readin of an oracle takes *O*(*m*) steps. Assuming that we are now given the oracle for element *a*_*k*,*i*_, the operation procedure can be seen in Fig. 2. From *j* = 0 to *j* = *m*, the rotation is applied about axis-*y* on the two level degree of freedom sequentially, which are controlled by the control qubits 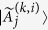. A total operation *U*^(*k*,*i*)^ takes *m* steps, which can be expressed as:


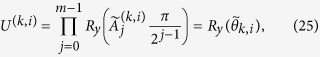


where 
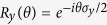
 and 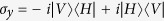. For input states |*V*, *k*, *i*〉, we obtain the output states


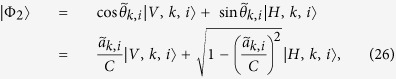


where 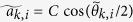.

For 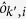 that represents *b*_*i*,*k*′_, we first perform a rotation on the input states 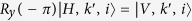. Then, following a similar procedure we obtain the output state


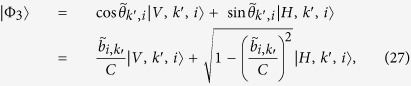


where 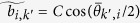.

Next we separate |*H*〉 and |*V*〉 into different paths, and combine the paths with |*V*〉 into |0, *i*〉, while leaving |*H*〉 in *k*, *i*〉 (|*k*′, *i*〉). Such procedure removes the path information of |*H*〉. For photonic systems, it can be simply realized with a non-polarizing beam splitter (NBS) and post-selecting^31^. Finally, we complete the oracle operation.

For large *m*, the error of the amplitude satisfies


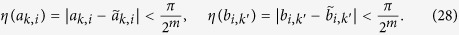


If *m* is infinitely large, the error can be infinitely small. Obviously, both time and spacial complexity the oracles are *O*(*m*).

## Additional Information

**How to cite this article**: Zhang, X.-D. *et al.* Quantum hyperparallel algorithm for matrix multiplication. *Sci. Rep.* **6**, 24910; doi: 10.1038/srep24910 (2016).

## References

[b1] Nielsen M. A. & Chuang I. L. Quantum Computation and Quantum Information (Cambridge University Press, Cambridge, England, 2000).

[b2] Shor P. Algorithms for quantum computation: Discrete logarithms and factoring. In Proceedings of the 35th Annual Symposium on Foundations of Computer Science (IEEE, New York, 1994), p. 124–134.

[b3] Grover L. A fast quantum mechanical algorithm for database search. in Proceedings of the 28th Annual ACM Symposium on Theory of Computing (ACM Press, New York, 1996), p. 212–219.

[b4] Long G. L. Grover algorithm with zero theoretical failure rate. Phys. Rev. A 64, 022307 (2001).

[b5] Toyama F. M., van Dijk W. & Nogami Y. Quantum search with certainty based on modified Grover algorithms: optimum choice of parameters. Quantum Inf. Process. 12, 1897 (2013).

[b6] Harrow A. W., Hassidim A. & Lloyd S. Quantum algorithm for linear systems of equations. Phys. Rev. Lett. 103, 150502 (2009). 19905613 10.1103/PhysRevLett.103.150502

[b7] Clader B. D., Jacobs B. C. & Sprouse C. R. Preconditioned quantum linear system algorithm. Phys. Rev. Lett. 110, 250504 (2013). 23829722 10.1103/PhysRevLett.110.250504

[b8] Le Gall F. & Nishimura H.. Quantum algorithms for matrix products over semirings. Algorithm Theory-SWAT p. 331 (2014)

[b9] Le Gall F. Quantum Algorithms for Matrix Multiplication. *in Proc. of SODA*’*12*, p. 1464.

[b10] Le Gall F. Quantum Algorithms for Matrix Multiplication. *in Proc. of ISAAC*’*12*, p. 639.

[b11] Aho A. V., Hopcroft J. E. & Ullman J. The design and analysis of computer algorithms. Addison-Wesley Longman Publishing Co., Boston, MA, 1974).

[b12] Buhrman H. & Spalek R. Quantum verification of matrix products. *in Proc. 17th ACM-SIAM Symposium on Discrete Algorithms*, p. 880. (2006).

[b13] Buhrman H., Cleve R., Watrous J. & de Wolf R. Quantum fingerprinting. Phys. Rev. Lett. 87, 167902 (2001). 11690244 10.1103/PhysRevLett.87.167902

[b14] Escartin J. C. G. & Posada P. C. Swap test and Hong-Ou-Mandel effect are equivalent. Phys. Rev A 87, 052330 (2013).

[b15] Lloyd S., Mohseni M. & Rebentrost P. Quantum algorithms for supervised and unsupervised machine learning. *arXiv*: 1307.0411.

[b16] Cai X. D. *et al.* Entanglement-Based Machine Learning on a Quantum Computer. Phys. Rev. Lett. 114, 110504 (2015). 25839250 10.1103/PhysRevLett.114.110504

[b17] Kiesel N. *et al.* Experimental analysis of a four-qubit photon cluster state. Phys. Rev. Lett. 95, 210502 (2005). 16384122 10.1103/PhysRevLett.95.210502

[b18] Walther P. *et al.* Experimental one-way quantum computing. Nature (London) 434, 169 (2005). 15758991 10.1038/nature03347

[b19] Leibfried D. *et al.* Creation of a six-atom ‘Schrodinger cat’ state. Nature (London) 438, 639 (2005). 16319885 10.1038/nature04251

[b20] Prevedel R. *et al.* High-speed linear optics quantum computing using active feed-forward. Nature (London) 445, 65 (2007). 17203057 10.1038/nature05346

[b21] Ren B. C. & Deng F. G. Hyper-parallel photonic quantum computation with coupled quantum dots. Sci. Rep. 4, 4623 (2014). 24721781 10.1038/srep04623 PMC3983618

[b22] Luo M. X. & Wang X. J. Parallel Photonic Quantum Computation Assisted by Quantum Dots in One-Side Optical Microcavities. Sci. Rep. 4, 5732 (2014). 25030424 10.1038/srep05732 PMC4101523

[b23] Ren B. C., Wang G. Y. & Deng F. G. Universal hyperparallel hybrid photonic quantum gates with dipole-induced transparency in the weak-coupling regime. Phys. Rev. A 91, 032328 (2015).

[b24] Barreiro J. T., Langford N. K., Peter N. A. & Kwiat P. G. Generation of hyperentangled photon pairs. Phys. Rev. Lett. 95, 260501 (2005). 16486324 10.1103/PhysRevLett.95.260501

[b25] Gao W. B. *et al.* Experimental demonstration of a hyper-entangled ten-qubit Schrodinger cat state. Nature Phys. 6, 331 (2010).

[b26] Sheng Y. B., Deng F. G. & Long G. L. Complete hyperentangled-Bell-state analysis for quantum communication. Phys. Rev. A 82, 032318 (2010).

[b27] Wang X. L. *et al.* Quantum teleportation of multiple degrees of freedom of a single photon. Nature (London) 518, 516 (2015). 25719668 10.1038/nature14246

[b28] Sheng Y. B. & Zhou L. Deterministic entanglement distillation for secure double-server blind quantum computation. Sci. Rep. 5, 7815 (2015). 25588565 10.1038/srep07815 PMC4295105

[b29] Sheng Y. B. & Zhou L. Deterministic polarization entanglement purification using time-bin entanglement. Laser Phys. Lett. 11, 085203 (2014).

[b30] Williams V. V. Multiplying matrices faster than Coppersmith-Winograd. *In Proc. of STOC12*, p. 887. (2012). probability distributions. *arXiv*: quant-ph/0208112.

[b31] Zhou X. Q., Kalasuwan P., Ralph T. C. & O’Brien J. L. Calculating unknown eigenvalues with a quantum algorithm. Nat. Photonics 7, 223 (2013).

